# Rapamycin extends life span of Rb1^+/−^ mice by inhibiting neuroendocrine tumors

**DOI:** 10.18632/aging.100533

**Published:** 2013-02-23

**Authors:** Carolina B. Livi, Rulon L. Hardman, Barbara A. Christy, Sherry G. Dodds, Diane Jones, Charnae Williams, Randy Strong, Alex Bokov, Martin A. Javors, Yuji Ikeno, Gene Hubbard, Paul Hasty, Zelton Dave Sharp

**Affiliations:** ^1^ Department of Molecular Medicine and Institute of Biotechnology, University of Texas Health Science Center, San Antonio, TX 78229; ^2^ Department of Radiology, University of Texas Health Science Center, San Antonio, TX 78229; ^3^ Department of Pathology, University of Texas Health Science Center, San Antonio, TX 78229; ^4^ Department of Pharmacology, University of Texas Health Science Center, San Antonio, TX 78229; ^5^ Department of Epidemiology & Biostatistics, University of Texas Health Science Center, San Antonio, TX 78229; ^6^ Department of Psychiatry, University of Texas Health Science Center, San Antonio, TX 78229; ^7^ Barshop Institute for Longevity and Aging Studies, University of Texas Health Science Center, San Antonio, TX 78245; ^8^ Cancer Therapy and Research Center, University of Texas Health Science Center, San Antonio, TX 78229; ^9^ Geriatric Research, Education and Clinical Center, Research Service, South Texas Veterans Health Care System, San Antonio, TX 78229

**Keywords:** mTOR, rapamycin, Rb1, neuroendocrine tumors

## Abstract

Chronic treatment of mice with an enterically released formulation of rapamycin (eRapa) extends median and maximum life span, partly by attenuating cancer. The mechanistic basis of this response is not known. To gain a better understanding of these *in vivo* effects, we used a defined preclinical model of neuroendocrine cancer, *Rb1^+/−^* mice. Previous results showed that diet restriction (DR) had minimal or no effect on the lifespan of *Rb1^+/−^* mice, suggesting that the beneficial response to DR is dependent on pRb1. Since long-term eRapa treatment may at least partially mimic chronic DR in lifespan extension, we predicted that it would have a minimal effect in *Rb1^+/−^* mice. Beginning at 9 weeks of age until death, we fed *Rb1^+/−^* mice a diet without or with eRapa at 14 mg/kg food, which results in an approximate dose of 2.24 mg/kg body weight per day, and yielded rapamycin blood levels of about 4 ng/ml. Surprisingly, we found that eRapa dramatically extended life span of both female and male *Rb1^+/−^* mice, and slowed the appearance and growth of pituitary and decreased the incidence of thyroid tumors commonly observed in these mice. In this model, eRapa appears to act differently than DR, suggesting diverse mechanisms of action on survival and anti-tumor effects. In particular the beneficial effects of rapamycin did not depend on the dose of *Rb1*.

## INTRODUCTION

Age is by far the biggest independent risk factor for a wide range of intrinsic diseases [1], including most types of cancer [2]. The age-adjusted cancer mortality rate for persons over 65 years of age is 15-times greater than for younger individuals [3]. Numerous studies demonstrate that age retarding interventions reduce cancer by decreasing incidence and/or severity (Reviewed in [4]). Diet restriction (DR) has a long history of retarding cancer [5] and most of the other age-associated diseases [6], consistent with life span extension in a wide range of organisms [7]. Genetic interventions resulting in pituitary dwarfism in mice, which causes growth factor reduction (GFR) and a reduction in associated signaling, also result in maximum lifespan extension [8], with a concomitant reduction in cancer severity [9, 10]. Thus, factors that inhibit growth appear to extend life span and reduce cancer.

mTORC1 (mechanistic Target of Rapamycin Complex 1) is central to cell growth by integrating upstream signals that include nutrients, growth factors and energy levels with stress responses for regulated cell growth. Thus, chronic mTORC1 inhibition could act similarly to DR and GFR. Supporting this possibility, the mTOR inhibitor rapamycin, increases life span in a variety of organisms including yeast [11], nematodes [12] and flies [13]. Using a chow containing a novel formulation of enterically delivered rapamycin (eRapa [14]), the NIA Intervention Testing Program [15] reported that long-term treatment extends both median and maximum lifespan of genetically heterogeneous mice (UM-HET3), even when started in late adulthood (20 months of age) [16], or at 9 months of age [17]. eRapa is the first drug reported to be capable of extending both median and maximum lifespan.

One explanation for the lifespan enhancement by eRapa is that chronic mTOR inhibition delays the onset and growth of neoplasms. Indeed, chronic eRapa (2.24 mg/kg/day diet) treatment reduced the incidence of lymphoma and hemangiosarcoma (two major cancers in the genetically heterogeneous mice studied by the ITP), and increased the mean age at death due to liver, lung and mammary tumors [16, 17]. Alternate possibilities are that the immune systems of treated mice better defend against their cancers or that the mice simply tolerate them longer. What is the basis of eRapa's ability to reduce cancer, and how does it compare to DR?

To gain an understanding of how chronic eRapa treatment compares with DR and affects cancer development, growth and progression, we used a mouse model deficient in the prototypical tumor suppressor, *Rb1*. Rb1 regulates cell cycle checkpoints for differentiation and in response to stress and is important for genome maintenance [18]. *Rb1*^+/−^ mice are highly predisposed to cancers of neuroendocrine origin [19] including pituitary (intermediate and anterior lobe), thyroid C-cell (which can metastasize to lung), and adrenal. Tumorigenic cells form after losing the remaining functional copy of the Rb1 tumor suppressor gene. The complete penetrance of tumor formation, growth and progression results in a short lifespan for *Rb1^+/−^* mice, which, unlike wild type mice, is minimally affected by diet restriction [20]. If eRapa acts in a similar manner to DR [16], we predicted that chronic eRapa treatment of *Rb1*^+/−^ mice would also have minimal effects on tumor development, growth, progression and life span. Surprisingly we find that eRapa treatment has a dramatic and positive effect on life span in both sexes of *Rb1*^+/−^ mice, which is associated with slower tumor development and growth.

## RESULTS

To address the question of whether eRapa mimics DR in mice deficient for a prototypical tumor suppressor gene function, we initiated chronic treatment by feeding randomly grouped males and females chow that included either eRapa at the concentration previously shown to extend life span (14 mg/kg food), [16, 17] or Eudragit (empty capsule control). Treatment was begun at approximately 9 weeks of age (>80% of animals started between 8-10 weeks (minimum at 7 weeks and maximum at 12 weeks, Table S1). Mice continued on these diets for the remainder of their lives. Based on the average amount of chow consumed per day, this delivers an approximate rapamycin dose of 2.24 mg/kg body weight/day [16]. Blood levels of rapamycin (determined by a mass spectrometry) averaged 3.9 ng/ml for males, 3.8 ng/ml for females for *Rb1*^+/−^ mice and 3.4 for males and 4.6 ng/ml for females for *Rb1*^+/+^ mice (Figure S1). Hematocrits were performed on blood from *Rb1*^+/+^ mice between 18 and 24 months of age and readings indicated normal values for mice (between 40 and 49%), indicating that long-term eRapa treatment does not adversely effect red blood cell production (data not included).

### eRapa extended life span of Rb1^+/−^ mice

**Figure 1:**
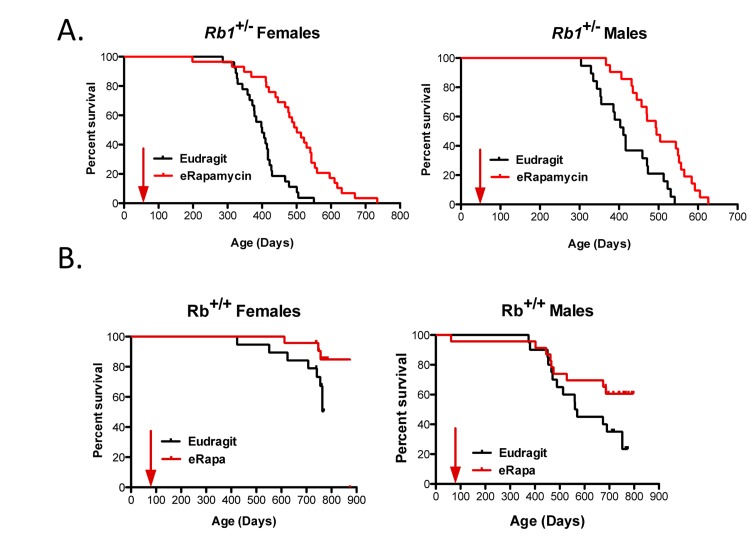
Survival plots for male and female *Rb1*^+/−^ (**A**) and *Rb1*^+/+^ (**B**) mice, comparing control-fed mice to those fed eRapa in the diet starting at approximately 9 weeks of age (indicated by arrow). Control (black line) and eRapa (red line) survival curves are shown. The horizontal axes represent life span in days and the vertical axes represent survivorship. *Rb1*^+/−^ mice obtained from the NCI Mouse Repository were bred by the Nathan Shock animal core to obtain the cohorts of male and female mice used in this study. Genotype was confirmed as previously described [20]. eRapa mice were fed microencapsulated rapamycin-containing food (14mg/kg food designed to deliver approximately 2.24mg of rapamycin per kg body weight/day that achieved about 4 ng/ml blood [14]. Diets were prepared by TestDiet, Inc., Richmond, IN using Purina 5LG6 as the base [14]. Control diet was the same but with empty capsules. P values in (**B)** were calculated by the log-rank test.

Unlike most mouse models of cancer [5], 50% DR had little (if any) effect on the development, growth and progression of neuroendocrine tumors or on life span of*Rb1*^+/−^ mice [20]. Since rapamycin has been predicted to act in a similar way to DR [16], we investigated if eRapa would also have little effect in this model. In stark contrast to DR, Figure 1A shows that *Rb1*^+/−^ males and females derive a significant longevity benefit from chronic treatment with eRapa. The Eudragit control-fed mice had a shorter mean life span than the eRapa-fed cohort for both females (377.5 versus 411 days) and males (mean age is 368.8 versus 419.8 days). Sex did not modulate the effect of eRapa on *Rb1*^+/−^ animals (Table 1).

**Table 1:** eRapa effects on survival of *Rb1*^+/−^ mice

	Coefficient	Hazard Ratio	SE	z	P
**eRapa**	−1.3177	0.2678	0.2400	−5.4909	0.00000004
**Sex**	0.1693	1.1844	0.2144	0.8005	0.42344718

Male and female *Rb1*^+/+^ littermates of the *Rb1*^+/−^ mice were also fed eRapa or control diets to ensure that this particular mutant strain (with a C57BL/6 background) is responsive to rapamycin. Once all *Rb1*^+/−^ mice had died and the effects of eRapa were evident, the *Rb1*^+/+^ littermates were euthanized. At this time, as expected, eRapa improved survival for both male and female *Rb1*^+/+^ mice as well (Fig. 1B). Similar to the previous results from the Intervention Testing Program eRapa experiments [16, 17], lifespan was extended more in females than in males (Table 2) in wild type (WT) littermates.

**Table 2:** eRapa effects on survival of *Rb1*^+/+^ mice.

	Coefficient	Hazard Ratio	SE	z	P
**eRapa**	−0.9305	0.3943	0.3631	−2.5625	0.01039082
**Sex**	−1.2818	0.2775	0.3840	−3.3382	0.00084312

### eRapa effects on tumor incidence at the end of life

At necropsy, *Rb1*^+/−^ mice were evaluated for the presence of neuroendocrine tumors and lung metastases. As shown in Table 3, there were no differences in the eRapa and Eudragit control groups in terms of presence of pituitary tumors (although we did observe a delay in their detection and reduction in size by magnetic resonance imaging (MRI), discussed below). We did observe a decreased incidence of thyroid C-cell carcinomas in the eRapa treated group of *Rb1*^+/−^ mice (p = 0.0112). Except for the modest decrease in thyroid tumors, this tumor spectrum is similar to *Rb1* heterozygotes treated with DR compared to those fed *ad libitum* [20]. Along with the decrease in thyroid C-cell tumors, eRapa also tended to reduce the incidence and severity of C-cell lung metastases (Table 4). Thus mice have a decreased cancer burden and live with tumors longer.

**Table 3:** Pathology of *Rb1*^+/−^ mice at necropsy.

	Eudragit	eRapa
**Tumor Incidence**		
Pituitary	97.5% (40)	100%^a^ (39)
Thyroid	90.0% (40)	66.7%^b^ (39)
Thyroid with lung metastases	37.5% (40)	28.2%^c^ (39)
Thyroid with adrenal metastases	2.5% (40)	7.7%^d^ (39)
Adrenal	30.0% (40)	23.1%^e^ (39)

a p = 0.9858,

b p = 0.0112;

c p = 0.3859;

d p = 0.5472,

e p = 0.4925

Two tailed, unpaired t test, GraphPad Prism.

**Table 4:** Incidence and pathology of *Rb1*^+/−^ lung metastases

	Eudragit		eRapa	
	Males	Females	Males	Females
**Grade**				
0	6	6	5	11
1	1	1	1	3
2	3	7	1	2
3	1	1	1	2
4	0	1	0	0
Total (Gr 1-4)	5	10	4	7

### eRapa delayed tumor development and slowed growth

Is delayed and/or reduced tumor growth the basis of life span extension by eRapa in this model? To address this question, we took advantage of the synchronous (spatial and temporal) development of tumors in this model Nikitin et al. [19, 21]. *Rb1*-deficient cells are first identified as atypical proliferates in the intermediate and anterior lobes of the pituitary, thyroid and parathyroid glands and the adrenal medulla at about 12 weeks of postnatal development. Atypical proliferates eventually form gross tumors with varying degrees of malignancy by postnatal day 350. Since we started treatment at around 8 weeks of age, eRapa might have an effect on the initiating events leading to loss of heterozygosity and/or subsequent formation of atypically proliferating cells. Perhaps more likely, eRapa slows growth and development of proliferates to gross tumors, which had probably begun at or around the time treatment was started. To test this latter possibility, we used MRI to follow pituitary and thyroid tumor development and growth in a subset of eRapa-treated*Rb1*^+/−^ mice (8 mice per treatment group were imaged between 1 and 4 times up to twice a month). MRI is well suited for following head and neck tumors that correspond to the primary tumor types *Rb1*^+/−^ mice develop. An initial cohort was used to identify the best timeframe for MRI scans. For this, 6 female *Rb1*^+/+^ mice (3 per group) and 10 *Rb1*^+/−^ mice (3 per group in males and 2 per group in females) were imaged in a single session or with 2 serial scans. This study indicated the ideal timeframe to image pituitary tumors was a window between 9 and 12 months of age, which covers the time from initial detection through monitoring tumor growth.

**Figure 2:**
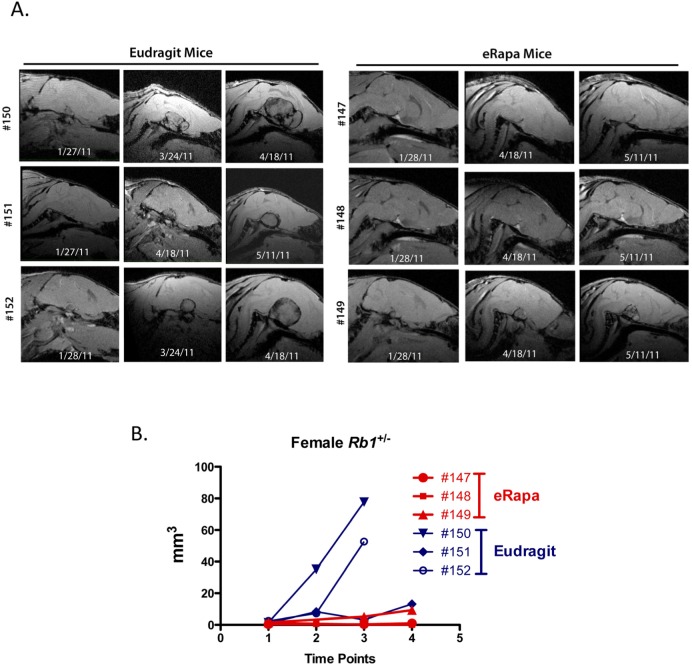
Effects of eRapa on pituitary and thyroid tumor development and growth. To identify effects on tumors, we used MRI as a non-invasive method to longitudinally monitor individual Rb^+/−^ mice. High-resolution images were obtained on a very high field strength Bruker Pharmascan 7.0T animal MRI scanner using a coil to focus on pituitary and thyroid tumors. Images were acquired using a spoiled gradient echo named Fast low angle shot MRI (FLASH) on the scanner. Images were acquired to yield predominantly T1 weighted contrast with TE (echo time) 4.5 msec, TR (repetition time) 450 msec, FA (Flip angle) 40 degrees, FOV (field of view) 20 x 20 mm, in plane spatial resolution 0.078 x 0.078 mm. Tumor volume was determined for each time point. (**A**) Serially acquired MRI images from eRapa and Eudragit-fed control mice at 9, 11 and 12 months of age. (**B**) Tumor volumes calculated from MRI image stacks at each time point comparing individual mice at multiple ages. Tumors in two of the Eudragit-fed (control) mice are detected earlier and grow faster than the 3 eRapa-fed mice.

Age matched *Rb1*^+/−^ females (3 per group) were scanned using MRI at 9, 11 and 12 months of age (Figure 2A shows sagittal plane sections of the serially acquired MRI images through the pituitary of eRapa and Eudragit treated mice). Calculated volumes based on the MRI image stacks (analyzed blind by a single radiologist, RLH) were plotted versus age at the date of imaging. In concert with extended longevity, the detection of pituitary tumors was delayed with a decrease in their growth in the eRapa-treated mice. Figure 2B shows that eRapa delayed development and/or reduced tumor growth at each time point when mice were imaged. More *Rb1*^+/−^ mice had detectable tumors identified during two separate MRI imaging sessions from the Eudragit control cohort (4 pituitary and 2 thyroid tumors out of 8 mice in March 2011 scan and 7 pituitary and 4 thyroid tumors out of 8 mice in April 2011 scan) compared to the mice eRapa-fed cohort (1 pituitary and 0 thyroid tumors out of 8 mice in March 2011 scan and 2 pituitary and 3 thyroid tumors out of 8 mice in April 2011 scan). Longitudinal monitoring allowed us to conclude that chronic rapamycin delays both the development of visible tumors and inhibited the growth of tumors once they were present.

## DISCUSSION

In mice, pRb1 is critical for DR-mediated lifespan extension [20], but not rapamycin-mediated life span extension. It is unclear why this is the case, since both of these interventions chronically inhibit mTORC1 [22]. However, differences in the downstream *in vivo* effects of DR and rapamycin have been previously reported [22]. As previously described by Harrison et al. [16], a distinguishing feature of eRapa is its ability to extend median and maximum life when the intervention starts at a relatively old age (600 days) in mice. By comparison, DR in most [23] but not all [24] reports shows little if any longevity benefit when started after 550 days of age (equivalent to 60 human years). DR started at 6 weeks of age reduced body growth for *Rb1*^+/−^ mice but did not affect growth of *Rb1*^−/−^ tumors [20]. In contrast to DR, chronic eRapa treatment did not affect body weight of *Rb1*^+/−^ mice (Livi et al., in preparation), but did reduce tumor growth. Previous studies in fruit flies show that rapamycin extends life span through a mechanism that is at least partly independent of TOR [13]. Consistent with those results, we find that eRapa, but not DR, extended life span and reduced the growth of neuroendocrine tumors in the *Rb1*^+/−^ model. It will be interesting to determine if pRb1 might be at least partially involved in those settings where responses to chronic eRapa and DR diverge.

**Figure 3:**
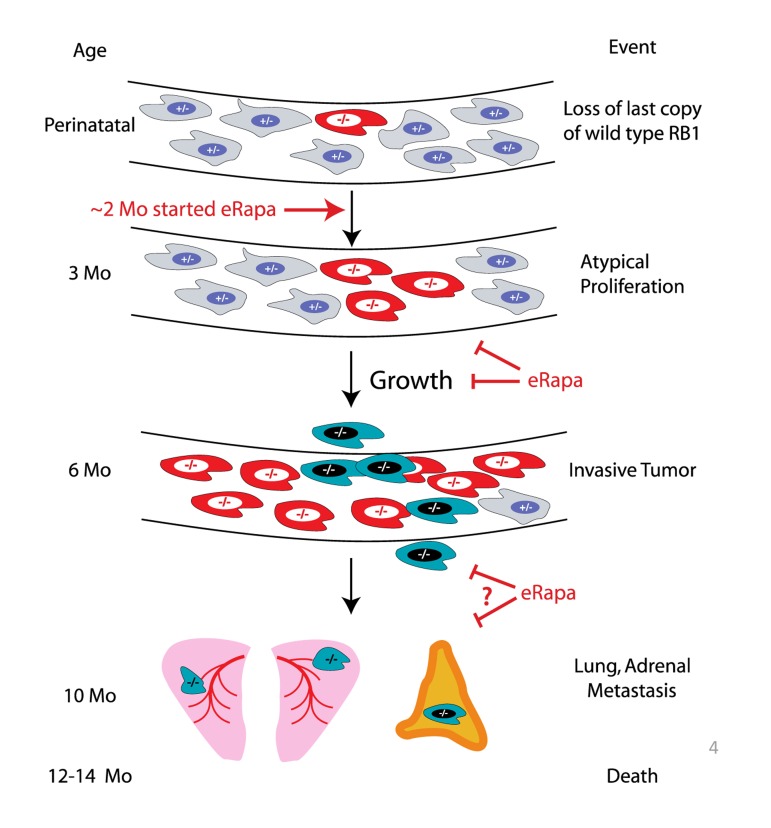
Summary of eRapa effects in the *Rb1*^+/−^ model of neuroendocrine tumorigenesis. Our MRI data are consistent with a delay of tumor development perhaps by inhibition of atypical proliferates and reduction in tumor growth. eRapa may inhibit lung metastasis and slow their growth.

Based on the longitudinal imaging data acquired by MRI (Figure 2), eRapa appears to inhibit *Rb1*^−/−^ pituitary tumor development and growth in *Rb1*^+/−^ mice (summarized in Figure 3), which is likely a major factor in its ability to extend lifespan in this model. Since we started eRapa at between 2 and 3 months of age, it would be interesting to know if it affects loss of heterozygosity (LOH) (Figure 3) in neuroendocrine tissues. The significant reduction in the incidence of thyroid C-cell carcinoma at necropsy in eRapa treated *Rb1^+/−^* mice (Table 3) also likely contributes to extended longevity. We also observed an apparent lessening of severity in lung metastases (Table 4), but this may be due to overall reduction of C-cell carcinomas. Metastasis of these to tumors to the adrenal (Table 3) has, to our knowledge, not been previously reported. A recent report linked an increase in metastasis with RAD001 treatment in a rat model of transplanted neuroendocrine tumors, which the authors attributed to alternations in tissue immune microenvironment [25]. Since RAD001 treatment was started subsequent to tumor implantation, it might be interesting to test this model in a prevention rather than treatment setting.

Two reports have linked pRb1 and mTOR. A genetic study in *D. melanogaster* established synergy between deletion of mTOR and pRb1 using an *in vivo* synthetic lethality screen of *Rb*-negative cells [26]. These authors found that inactivation of *gig* (fly TSC2) and *rbf* (fly Rb) is synergistically responsible for oxidative stress leading to lethality. In a separate study, El-Naggar et al., [27] found that loss of the *Rb1* family (Rb1, Rbl1 and Rbl2) in primary cells derived from triple-knockout mice led to overexpression of mTOR and constitutive phosphorylation of Ser473 on Akt , which is oncogenic. The inhibition of tumor development and growth in *Rb1*^+/−^ mice by eRapa is also consistent with a recent report showing that mTOR inhibition partially alleviated tumor development in an *Rb*^F/F^;K14creER™; *p107*^−/−^ model of squamous cell carcinoma [28], and with several reports demonstrating the effectiveness of rapamycin in mouse cancer models for tumor reduction and life span extension [29-31]. Potential mechanism may be by way of indirect effects or rapamycin on the tumor microenvironment [32] and/or senescent cells [33].

The reduction in lung metastases is consistent with ribosome profiling that revealed transcript-specific translational control mediated by oncogenic mTOR signaling, including a distinct set of pro-invasion and metastasis genes [34]. It will be interesting to determine whether chronic eRapa treatment affects these genes in thyroid C-cell neoplasms. We also observed metastasis of thyroid tumors to adrenal glands, albeit at a low frequency but eRapa treatment did not effect.

Neuroendocrine tumors are unique in their ability to secrete hormones or deleterious bioactive products [35]. It was previously reported that the rapalog Everolimus (RAD-001) in combination with ocreotide lanreotide (compared to placebo) improved the clinical picture of carcinoid patients by reducing circulating chromogranin A and 5-hydroxyindoleacetic acid, two tumor-secreted bioactive products responsible for some of the symptoms [36]. Thus, another potential mechanism for life span extension in *Rb1*^+/−^ mice by eRapa could be due the prevention of the production and/or secretion of hormones or deleterious bioactive factors.

*Rb1* is known to have an important role in somatic growth regulation, since increased *RB1* dose reduced animal size [37]. Determining if there is a link between *Rb1* (a negative regulator of growth) and mTORC1 (a positive regulator of growth) in growth of tumors could suggest new therapeutic and prevention targets for drug development. One prediction is that mice over expressing pRb1 will have decreased mTOR activity and be long lived through prevention, delay or a reduction in severity of age-related diseases.

Here we show that eRapa extends the life span for *Rb1*^+/−^ mice. We find eRapa-fed mice exhibit a delay in the onset and/or progression of neuroendocrine tumors. These results are in direct contrast with DR. Thus, mTORC1 inhibition and DR likely use different modes for life span extension.

## METHODS

### Mice and life span

Mice (strain B6.129S2(Cg)-Rb1^tm1Tyj^) for breeding were obtained from the NCI MMHCC Repository. Although they have similar phenotypes, the strain used in the diet restriction study by Sharp et al., [20] was different having been generated by Lee et al [38]. The procedures and experiments involving use of mice were approved by the Institutional Animal Care and Use Committee and are consistent with the NIH Principles for the Utilization and Care of Vertebrate Animals Used in Testing, Research and Education, the Guide for the Care and Use of Laboratory Animals and the Animal Welfare Act (National Academy Press, Washington, DC). Genotyping was done as described previously [20]. Cohorts of mice were fed microencapsulated rapamycin-containing food (14 mg/kg food designed to deliver ~2.24 mg of rapamycin per kg body weight/day to achieve about 4 ng/ml of rapamycin per kg body weight/day) prepared by TestDiet, Inc., Richmond, IN using Purina 5LG6 as the base [14]. Control diet was the same but with empty capsules.

### Rapamycin food concentration

Rapamycin was quantified in food using HPLC with tandem mass spectrometry detection. Briefly, 100 mg of food for spiked calibrators and unknown samples were crushed with a mortar and pestle, then vortexed vigorously with 10 μL of 250 μg/mL ASCO (internal standard) and 4.0 ml of mobile phase A. The samples were then mechanically shaken for 10 min, centrifuged for 10 min, and then centrifuged in microfilterfuge tubes for 1 minute. Ten μL of the final extracts was injected into the LC/MS/MS. The ratio of the peak area of rapamycin to that of the internal standard (response ratio) was compared against a linear regression of calibrator response ratios at rapamycin concentrations of 0, 2, 5, 10, 30, and 60 ng/mg of food to quantify rapamycin. The concentration of rapamycin in food was expressed as ng/mg food (parts per million).

### Rapamycin blood measurements

Measurement of rapamycin used HPLC-tandem MS. RAPA and Ascomycin (ASCO) were obtained from LC Laboratories (Woburn, MA). HPLC grade methanol and acetonitrile were purchased from Fisher (Fair Lawn, NJ). All other reagents were purchased from Sigma Chemical Company (St. Louis, MO). Milli-Q water was used for preparation of all solutions. RAPA and ASCO super stock solutions were prepared in methanol at a concentration of 1 mg/ml and stored in aliquots at −80°C. A working stock solution prepared each day from the super stock solutions at a concentration of 10 μg/ml was used to spike the calibrators.

Calibrator and unknown whole blood samples (100 μL) were mixed with 10 μL of 0.5 μg/mL ASCO (internal standard), and 300 μL of a solution containing 0.1% formic acid and 10 mM ammonium formate dissolved in 95% HPLC grade methanol. The samples were vortexed vigorously for 2 min, and then centrifuged at 15,000 *g* for 5 min at 23°C (subsequent centrifugations were performed under the same conditions). Supernatants were transferred to 1.5 ml microfilterfuge tubes and centrifuged at 15,000 *g* for 1 min and then 40 μL of the final extracts were injected into the LC/MS/MS. The ratio of the peak area of rapamycin to that of the internal standard ASCO (response ratio) for each unknown sample was compared against a linear regression of calibrator response ratios at 0, 1.25, 3.13, 6.25, 12.5, 50, and 100 ng/ml to quantify rapamycin.

The HPLC system consisted of a Shimadzu SCL-10A Controller, LC-10AD pump with a FCV-10AL mixing chamber (quarternary gradient), SIL-10AD autosampler, and an AB Sciex API 3200 tandem mass spectrometer with turbo ion spray. The analytical column was a Grace Alltima C18 (4.6 x 150 mm, 5 μ) purchased from Alltech (Deerfield, IL) and was maintained at 60°C during the chromatographic runs using a Shimadzu CTO-10A column oven. Mobile phase A contained 10 mM ammonium formate and 0.1% formic acid dissolved in HPLC grade methanol. Mobil phase B contained 10 mM ammonium formate and 0.1% formic acid dissolved in 90% HPLC grade methanol. The flow rate of the mobile phase was 0.5 ml/min. Rapamycin was eluted with a step gradient. The column was equilibrated with 100% mobile phase B. At 6.10 minutes after injection, the system was switched to 100% mobile phase A. Finally, at 15.1 min, the system was switched back to 100% mobile phase B in preparation for the next injection. The rapamycin transition was detected at 931.6 Da (precursor ion) and the daughter ion was detected at 864.5 Da. ASCO was detected at 809.574 Da and the daughter ion was 756.34 Da.

### Survival Analysis Methods

An entry for each mouse in the study was created in a database used by the Nathan Shock Animal core. The age at which each animal died was recorded. Survival durations for animals that either lived past the end-date of the study, were terminated, or died accidentally were treated as right-censored events. Cox proportional hazard models [39] were fitted to the wild type and *Rb1*^+/−^ subsets of the data, with eRapa and gender as additive predictor variables. Some animals were transferred to a different facility part-way through their life spans so the final facility at which they were housed was also added to the Cox models, as a stratifying variable. The R statistical language was used for the analysis [40, 41]. The mice in the life span studies were allowed to live out their life span, i.e., there was no censoring due to morbidity in the groups of mice used to measure lifespan of *Rb1*^+/−^ mice. Mice were euthanized only if they were either [1] unable to eat or drink, [2] bleeding from a tumor or other condition, or [3] when they were laterally recumbent, i.e., they fail to move when prodded or are unable to right themselves.

### MRI Methods

Images were acquired on a Bruker Pharmascan 7.0T MRI scanner. Images were obtained in the sagittal plane through the brain and coronal plain through the neck (focused on the thyroid gland) using 2D spoiled gradient echo technique to quickly obtain high-resolution images (fast low angle shot magnetic resonance imaging - FLASH on our scanner). FLASH protocol was TE/TR 5 msec/450msec, Averages 1, Flip angle 40 deg, Field of view 20 mm x 20 mm, matrix size 256x256, In plane resolution was 0.078 x 0.078 mm, slice thickness 0.5 mm. The FLASH sequence shows predominantly T1 weighted image contrast. A single blinded radiologist (RLH) evaluated images for the presence and tumor volume used to plot detection and growth data. Images were analyzed using an open source image processing software, OsiriX, version 2.7.5. The pituitary gland was identified on all images and volume was calculated by measuring the greatest anterior-posterior, cranial-caudal, and right-left length. Volumes were then determined using prolate ellipse formula. Data were then parsed by treatment group and plotted in Prism (GraphPad).

### Procedures for examination of pathology in mice

Fixed tissues (in 10% neutralized formalin) were embedded in paraffin, sectioned at 5 μm, and stained with hematoxylin-eosin. Diagnosis of each histopathological change was made using histological classifications for aging mice as previously described [9, 20, 42, 43].

### Pathology assessments

A list of lesions was compiled for each mouse. The severity of neoplastic lesions was assessed using the grading system previously described [9, 9, 20, 42, 43, 42, 43]. Two pathologists separately examined all of the samples without knowledge of their genotype or age. Briefly, lung pathology grade is based on the area of the lung section infiltrated by metastatic tumor tissue with 0 being no tumor cells observed and 4 being the largest area taken by tumor.

## SUPPLEMENTARY DATA


