# Supplementary material for: Rapamycin extends life span of Rb1+/− mice by inhibiting neuroendocrine tumors
Source: Aging (Albany NY). 2013 Feb 23;5(2):100–10. doi: 10.18632/aging.100533 (PMC3616197; doi:10.18632/aging.100533)
Supplement: Supplementary file 1 [file aging-05-100-s001.pdf]

SUPPLEMENTARY DATA

Table S1. Age (weeks) at treatment initiation

	Rb1$^{+/-}$		*Rb1*$^{+/+}$	
**Diet Start Range**	**#Mice**	**%**	**# Mice**	**%**
7-8	7	7.2	11	11.2
8-9	49	50.5	47	48.0
9-10	40	40.3	39	48.0
12	1	1.0	1	1.0
**Average**	8.9		8.8	
**Youngest**	7.0		7.0	
**Oldest**	12.0		12.0	

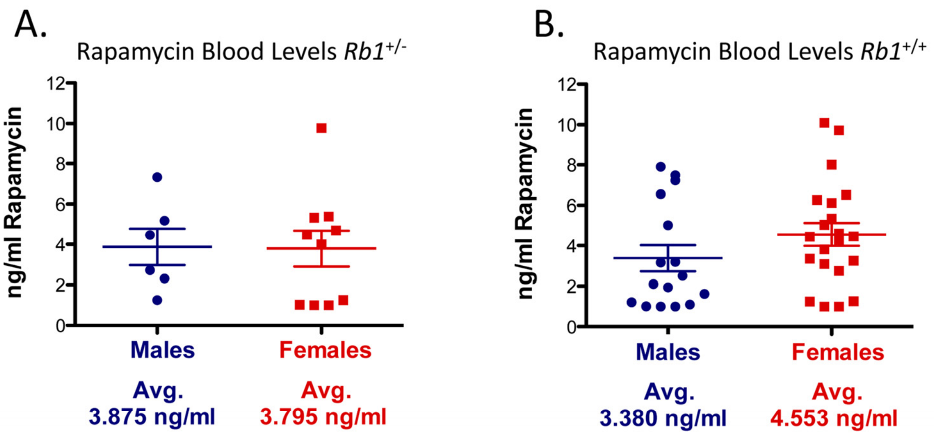

Figure S1. Rapamycin levels were quantified as described in Methods. The concentration of rapamycin was expressed as ng/ml of whole blood.
